# Partnership Satisfaction in Living Kidney Donors

**DOI:** 10.3389/fpsyt.2018.00353

**Published:** 2018-08-03

**Authors:** Mariel Nöhre, Iris Pollmann, Marie Mikuteit, Karin Weissenborn, Faikah Gueler, Martina de Zwaan

**Affiliations:** ^1^Department of Psychosomatic Medicine and Psychotherapy, Hannover Medical School, Hannover, Germany; ^2^Department of Neurology, Hannover Medical School, Hannover, Germany; ^3^Department of Nephrology, Hannover Medical School, Hannover, Germany

**Keywords:** kidney transplantation, living kidney donors, partnership satisfaction, quality of marriage index, partnership status

## Abstract

Due to organ shortage, living kidney donation is gaining increasing importance. Medical progress enables a successful transplantation between unrelated individuals, even individuals with AB0-incompatibilities. Spouses are the largest group of living kidney donors. The aim of this study was to assess partnership status and partnership satisfaction in living kidney donors. In the cross-sectional study we investigated 361 living kidney donors. The time since donation ranged between 1 and 38 years. The partnership satisfaction was assessed with the German version of the Quality of Marriage Index. We compared the donor sample with a representative German population sample (*n* = 1995). In addition, we compared donors who have donated to their partner (spouse donors) to those who have donated to someone else (non-spouse donors). In comparison to the population sample significantly more kidney donors were living in a relationship (82 vs. 60%). Most donors reported an unchanged (76.6%) or improved (20.5%) relationship to the recipient since transplantation. A significantly higher partnership satisfaction could be found in the donor sample compared to the population sample which was mainly due to a higher partnership satisfaction of the spouse donors compared to the non-spouse donors. High partnership satisfaction in living kidney donors might be an indicator for a successful selection process before transplantation. Alternatively, kidney donation might have a stabilizing or even positive impact on the partnership. Due to the design of our study causative interpretations cannot be made. Therefore, prospective studies are required to assess partnership satisfaction before and after living kidney donation.

## Introduction

Kidney transplantation is the preferential treatment for end-stage renal disease (ESRD) (1). A growing number of patients are on the waiting list for organ transplantation. In Germany about 8,000 patients are in need for a kidney. At the same time in 2017 post-mortal organ donations have reached the lowest level in 20 years (2). In Germany 9.7 donors per 1 million inhabitants have been registered in 2017 (2). This circumstance leads to an increasing importance of living kidney donation. In 2017, 1921 kidney transplantations were performed in Germany; 557 (29%) of the transplanted kidneys were donated by living donors (3). Several advantages are associated with living kidney donations such as shorter ischemia time, superior organ quality, and a longer graft survival (4). The highest advantage is of course that the recipient does not have to stay on dialysis for many years. Currently the waiting time for post-mortal donation lies on average between 6 and 7 years, whereas patients with blood group 0 often have to wait longer (5).

With the development of more effective immunosuppressive medication and an expanding experience regarding AB0-incompatible transplantation, also genetically unrelated living donors are able to donate a kidney (6, 7). In Germany, a close relationship between donor and recipient is mandatory. Altruistic or anonymous donation is legally not allowed. Spouses, which seem to be as suitable as other genetically unrelated living donors (7, 8), represent a large group of organ donors. Due to their close relationship to the recipient, often living in the same household, they experience the consequences of ESRD first hand. It is well-known that chronic illnesses do not only have an impact on the patients but their partners as well. Several studies suggest that the partnership can be affected by ESRD, leading to psychological distress also in the spouses of the chronically ill patient (9, 10). Partnership satisfaction is associated with mental as well as physical health (11). A recent study shows that partnership satisfaction is positively related to personal well-being (12). The kidney donation is a decisive event, which might lead to changes in several aspects of life. Therefore, it is important to take a closer look at the partnership satisfaction in the group of living kidney donors (LKD).

Previous research indicates that the partnership quality improves or remains the same after living kidney donation (8, 13, 14). A positive impact on the partnership quality could even be found when one partner donated for a mutual child (15) or to a close friend or relative (16). These findings indicate that living kidney donation most likely has a positive effect on partnership satisfaction. However, most of these results were based on small sample sizes (15, 16) or on investigator-generated questions and were not obtained with validated instruments (8, 13, 14). To our knowledge limited information exists about the quality of the relationship in LKD in comparison to a general population sample.

The aim of this study was therefore (1) to compare partnership status and partnership satisfaction between LKD and a representative population sample of the same age range and (2) to compare partnership satisfaction between donors having donated to their partner (spouse donors) with those having donated to someone else (non-spouse donors) using the German version of the Quality of Marriage Index (QMI-D) (17, 18). As most prior research suggests, we expected to find a high partnership satisfaction in couples after donation regardless of the recipient (8, 13–16).

## Methods

### Participants

#### Living kidney donors

**Figure 1:**
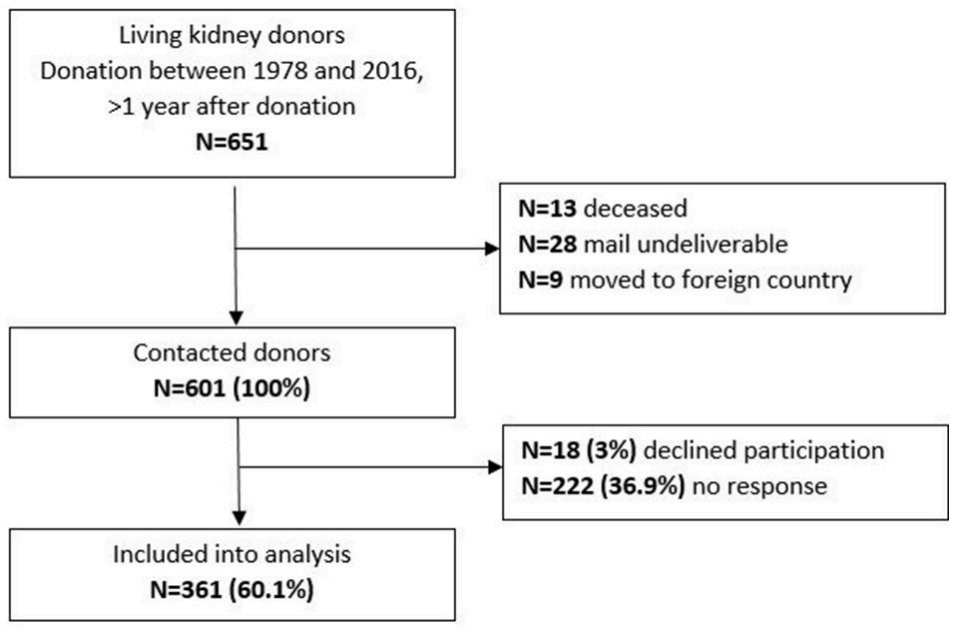
Study flow chart.

All individuals who were registered as LKD in the outpatient database of Hannover Medical School were contacted. The transplantation had to be at least 1 year ago. Therefore, individuals who had donated a kidney between 1978 and 2016 were included. Questionnaires and a cover letter explaining the study were mailed to them. They were asked to return the completed packages via addressed and pre-stamped envelopes. If there was no response the package was resent up to three times. In case packages were returned as undeliverable, the new address of the donors was identified with administrative assistance. Of the 651 donors of the database, 601 could be located (13 were deceased and in 37 no valid address could be identified) and 361 returned the survey. This corresponds to a response rate of 60.1% (Figure 1). The participating donors were aged between 29 and 79 years.

The Institutional Ethics Board of Hannover Medical School approved the study (no 3252-2016). All participants gave written consent and the returned surveys were de-identified before the statistical evaluation.

#### Population sample

As a control group a representative sample of the German population regarding age, gender, and educational level was used. The population sample was recruited by a demographic consulting company (USUMA, Berlin, Germany). Approval to conduct this study was given by the Ethics Committee of the University of Leipzig (Az 452-15-21122015). The participants had to speak German fluently and had to give written informed consent. They were chosen randomly in a three-stage sampling procedure. The selected individuals were approached face-to-face by a trained interviewer giving them information about the study. After informed consent was given sociodemographic data were assessed. Subsequently participants were asked to complete self-report questionnaires. A total of 2524 individuals participated. For comparison with the donor sample only participants from this survey aged between 29 and 79 years were selected (*n* = 1995).

### Assessment instruments

#### Quality of marriage index

The Quality of Marriage Index was published in 1983 by Norton et al. (18). It is a commonly used instrument to assess global partnership quality in individuals involved in steady partnerships. Despite the name of the questionnaire, the Quality of Marriage Index can be used to assess partnership satisfaction in unmarried couples as well (17). In this study the German version was used which has been validated by Zimmermann et al. (17). The self-report questionnaire consists of six items. The items are: “We have a good relationship.,” “My relationship with my partner is very stable.,” “My relationship with my partner is strong.,” “My relationship with my partner makes me happy.,” “I really feel like part of a team with my partner.,” and “All things considered, what degree of happiness best describes your relationship?” (18). The items 1 to 5 are rated on a 7-point Likert scale ranging from 1 (“strongly disagree”) to 7 (“strongly agree”). Item 6 is assessed on a 10-point Likert scale ranging from 1 (“very unhappy”) to 10 (“perfectly happy”). A total score of all items is calculated ranging between 6 and 45. A cutoff score of 34 or higher indicates a high satisfaction with the relationship. For the analyses the total score as well as the cutoff score was used as suggested by Zimmermann et al. (17). All participants who indicated that they were involved in a relationship were asked to fill out the QMI-D.

#### Sociodemographic and donation-specific variables

Investigator-generated questions were used to assess donor demographics and year of donation. In addition, donors were asked to rate changes in the relationship with the recipient since transplantation on a 5-point Likert scale between “very negative,” and “very positive.”

### Statistics

Statistical analyses were performed with IBM® SPSS® Statistics Version 24. Mann-Whitney U-tests were used for comparison of continuous data between the population sample and the donor sample, between spouse and non-spouse donors, and between sexes. Chi square tests were used for categorical data.

To adjust for differences in age and sex linear regression analyses with the QMI-D total score as the dependent variable and binary logistic regression analyses with partnership status as the dependent variable and age, sex and group (population vs. donors and spouse vs. non-spouse donors) as independent variables were conducted.

Eta squared (η^2^) and Cohen's *d* were calculated to estimate the effect size for the differences between QMI-D total scores. For η^2^ a value of 0.02 indicates a small effect, 0.13 a medium and 0.26 a large effect. For Cohen's *d* 0.2 indicates a small, 0.5 a medium, and 0.8 a large effect.

## Results

### Comparison between living kidney donors and the population sample

Results are summarized in Table 1. The donor sample differed from the population sample regarding age [median 57.0 (IQR 13) vs. 51.0 (IQR 20) years; *Z* = −6.564, *p* < 0.001], sex [females 60.9% vs. 51.3%; *X*^2^ = 7.510 (df = 1), *p* = 0.006], and educational level [≥12 years of education 28.0% vs. 21.7%, *X*^2^ = 4.625 (df = 1), *p* = 0.032].

**Table 1:** Comparison between population and donor sample and between spouse donors and non-spouse donors.

	**Population (29-79 years)**	**Living donors**			**Statistics (Mann-Whitney-U-tests and Chi-Square tests)**	
		**Total**	**Spouse donors**	**Non-spouse donors**	**Population vs. donors**	**Spouse donors vs. non-spouse donors**
N	1205	243	104	139	–	–
**Age, years**						
mean (*SD*)	51.6 (12.8)	57.2 (9.3)	57.8 (8.5)	56.8 (9.8)	*Z* = −6.564, *p* < 0.001	ns
median (IQR)	51.0 (20)	57.0 (13)	57.0 (12)	57.0 (14)		
**Sex, women** % (*n*)	51.3 (618)	60.9 (148)	69.2 (72)	54.7 (76)	*X*^2^ = 7.510 (df = 1), *p* = 0.006	*X*^2^ = 5.293 (df = 1), *p* = 0.021
**Educational level**, ≥12 years of education % (*n*)	21.7% (262)	28.0% (68)	20.2% (21)	33.8% (47)	*X*^2^ = 4.625 (df = 1), *p* = 0.032	*X*^2^ = 5.277 (df = 1), *p* = 0.022
**QMI-D, score**						
mean (SD)	38.9 (6.4)	40.4 (5.4)	41.4 (4.6)	39.7 (5.9)	Z = −3.296, *p* = 0.001^*^^#^	Z = −2.401, *p* = 0.016^*^^+^
median (IQR)	40.0 (7)	43.0 (6.0)	43.0 (6)	42.0 (6)	η^2^ = 0.014, Cohen's *d* = 0.24	η^2^ = 0.036, Cohen's *d* = 0.361
**QMI-D cutoff < 34**, % (*n*)	16.0 (193)	9.9 (24)	5.8 (6)	12.9 (18)	*X*^2^ = 5.984 (df = 1), *p* = 0.014	ns

* Differences between samples remained significant after adjusting for age, sex and educational level using linear regression analyses.

# The difference in QMI-D total scores was significant in both sexes: women (Z = −2.319, p = 0.020), men (Z = −2.550, p = 0.011)

+ The difference in QMI-D total scores was significant in female donors (Z = −2.95, p = 0.003) but not in male donors (Z = −0.175, p = 0.861)

Overall, QMI-D scores did not differ between women and men, neither in the population sample [women: m 38.6 (SD 6.4) men: m 39.4 (SD 5.9)] nor in the donor sample [women: m 39.9 (SD 5.9); men: m 41.1 (SD 4.4)].

**Figure 2:**
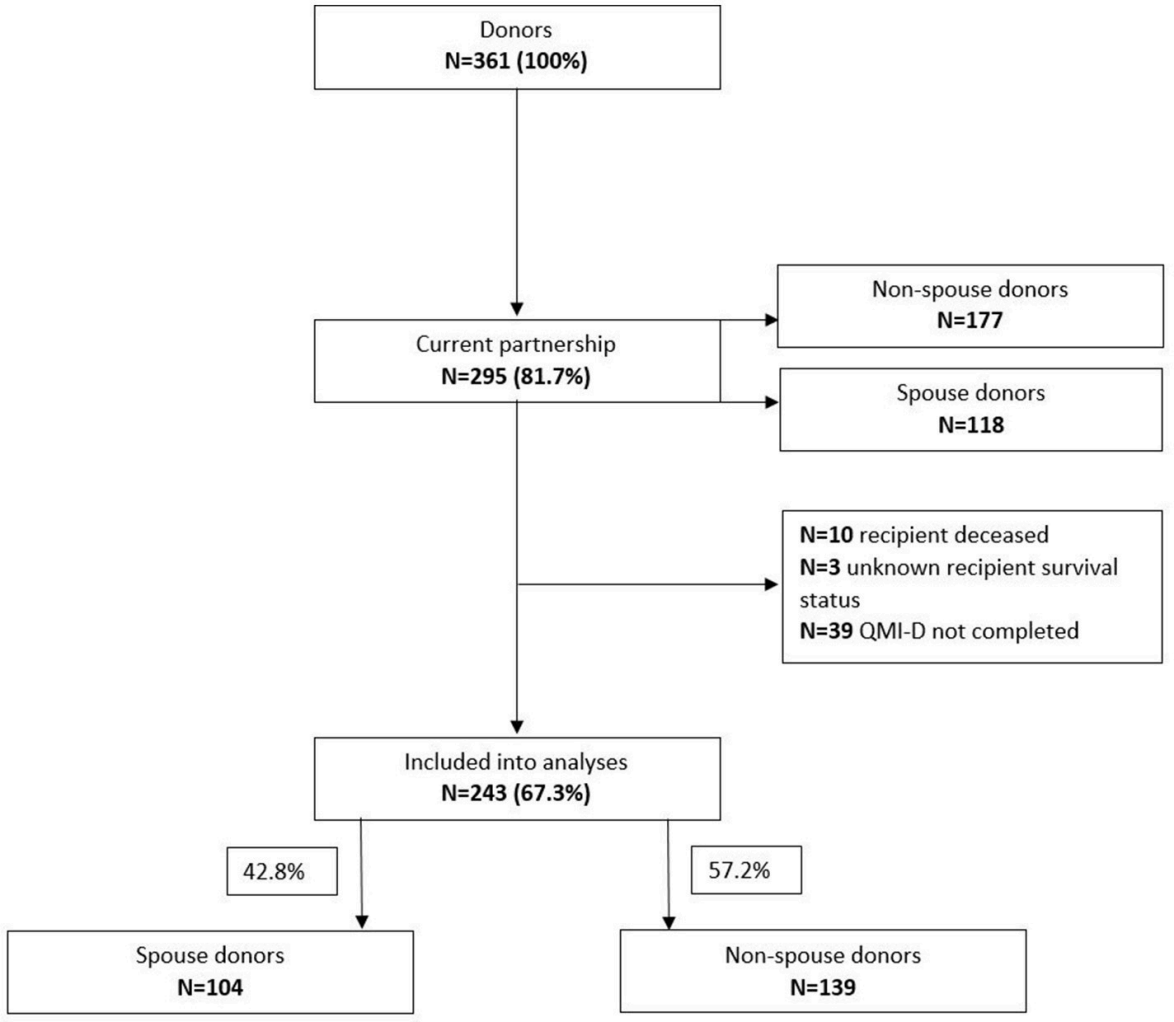
Partnership status in the living donor sample.

Two-hundred and ninety-five (81.7%) of the LKD in our sample indicated to be in a current partnership. Donors who did not complete the QMI-D and those whose recipient was deceased or had an unknown survival status were excluded; 243 remained for further analyses (Figure 2). Out of those, 104 (42.8%) had donated their kidney to their current partner (spouse donors) and 139 had donated to a different recipient (non-spouse donors) i.e., children or long standing friends.

In the population sample 1205 individuals aged between 29 and 79 years reported to be in a partnership (60.4%). Of those, all completed the QMI-D. The frequency of being in a partnership differed significantly from the LKD sample [81.7% vs. 60.4%; *X*^2^ = 60.0468 (df = 1), *p* < 0.01]. This was true for spouse donors (88.1%) [*X*^2^ = 40.831 (df = 1), *p* < 0.001] as well as non-spouse donors (78.3%) [*X*^2^ = 27.726 (df = 1), *p* < 0.001]. These differences remained significant after adjusting for age, sex, and educational level using binary logistic regression analyses.

QMI-D total scores were significantly higher in the donor sample [median 43.0 (IQR 6.0)] in comparison to the population sample [median 40.0 (IQR 7.0); Z = −3.296, *p* = 0.001]. Cohen's *d* = 0.24 indicated a small effect size. The difference remained significant after adjusting for age, sex, and educational level. The differences in QMI-D total scores between groups were significant in both sexes [women (Z = −2.319, *p* = 0.020), men (Z = −2.550, *p* = 0.011)].

QMI-D scores below 34 indicate an unhappy relationship. Significantly less unhappy relationships were reported in the donor sample compared with the population sample [9.9% vs. 16.0%, X^2^ = 5.984 (df = 1), *p* = 0.014].

### Comparison between spouse and non-spouse donors

Spouse donors and non-spouse donors did not differ regarding age. However, there were significantly more women in the spouse donor sample [69.2 vs. 54.7%, *X*^2^ = 5.293 (df = 1), *p* = 0.021]. The educational level was significantly higher in the non-spouse donors [≥12 years of education 33.8% vs. 20.4%, *X*^2^ = 5.277 (df = 1), *p* = 0.022].

Significantly more spouse donors reported to be in a relationship [88.1 vs. 78.3%; *X*^2^ = 5.395 (df = 1), *p* = 0.020]; this result remained significant after controlling for age, sex, and educational level.

Significantly higher QMI-D scores were found in the spouse donors compared to the non-spouse donors [median 43.0 (IQR 6) vs. 42.0 (IQR 6), Z = −2.401, *p* = 0.016]. The difference remained significant after adjusting for age, sex, and educational level. The effect size was small with a Cohen's *d* of 0.361. When analyzing the difference in QMI-D total scores separately for female and male spouse and non-spouse donors a significantly higher QMI-D total score was found in female spouse donors compared to female non-spouse donors (Z = −2.95, *p* = 0.003), but not between male spouse donors and male non-spouse donors.

No statistically significant difference between spouse and non-spouse donors could be found in the number of donors reporting a QMI-D score below the categorical cut-off of 34, indicating an unhappy relationship. Overall, the percentage of unhappy relationships was small in both groups (5.8% spouse donors vs. 12.9% non-spouse donors).

**Figure 3:**
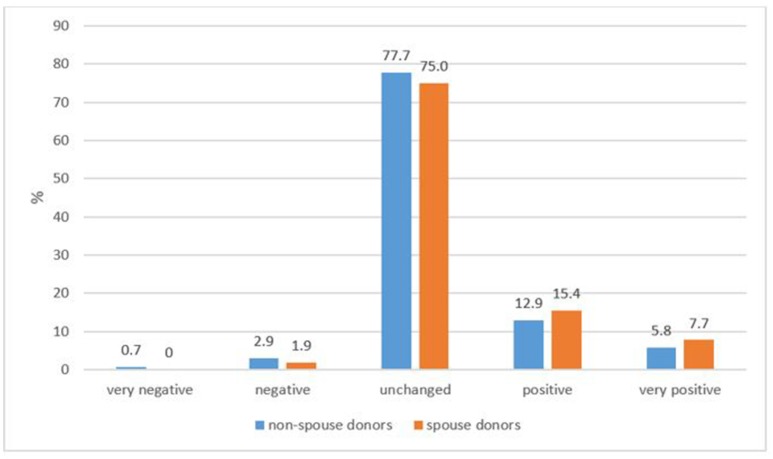
Change in the relationship since transplantation between donor and recipient (donor's perspective).

Overall, 97.1% of all donors reported an “unchanged” or “improved” relationship with the recipient; 98.1% of the spouse donors and 96.4% of the non-spouse donors (ns; Figure 3). However, a small group of donors reported a negative development of their relationship with the recipient, 3.6% of the non-spouse donors and 1.9% of the spouse donors.

## Discussion

The aim of this study was to assess partnership status and partnership satisfaction after living kidney transplantation from the donor's perspective with a well-validated instrument. In comparison to a representative German population sample it became apparent that significantly more participants in the donor sample were in a relationship regardless if they had donated to their spouse or another person. A high partnership frequency in the spouse donor sample was expected, as by definition 100% of the spouse donors were involved in a relationship by the time of donation. Interestingly, a significantly higher partnership frequency could also be found in the non-spouse donor sample compared to the population sample, with the difference remaining significant after adjusting for age and sex. A possible explanation might be—as suggested in the literature—that married individuals are healthier than unmarried individuals (19–21).

Two different hypotheses can be formulated trying to explain these results: On the one hand healthier individuals might have a higher chance to get married, on the other hand marriage might have a positive effect on health (21). Current research indicates that both assumptions might be correct, whereas causative relations still remain unclear (22). Regardless of causality, these results are in line with previous research showing that LKD are mentally and physically “healthier” than individuals in age-matched population samples and report higher health-related quality of life (HRQoL) (23). To understand these results one has to take into consideration that potential LKD have to undergo an extensive medical screening as well as a psychosocial evaluation. The medical screening serves the purpose to recognize medical circumstances which might put the donor or the recipient in danger. Underlying cardiovascular diseases (i.e., diabetes mellitus, coronary artery disease, severe hypertension and many other chronic diseases) are medical reasons precluding donation (24). The psychosocial evaluation is performed to assess the donor's motivation, knowledge about the transplantation process as well as psychosocial health (25). The aim of this rigorous selection process is to identify donors who are well informed regarding the transplantation and its risks, report comprehensible motivations and are physically and mentally stable. As a result, particularly “healthy” individuals, who statistically have a higher change of being married (19–21), are favored as potential donors, consequently leading to a selection bias.

In comparison to the population sample significant higher partnership satisfaction as well as fewer unhappy partnerships indicated by a QMI-D score below the categorical cut-off could be found in the donor sample. These results may be interpreted in the context of our previous study showing that LKD possess more adaptive personality traits in terms of higher agreeableness and lower neuroticism compared to a population sample (26). In a recent meta-analysis it could be shown that four personality characteristics, among others high agreeableness and low neuroticism correlate significantly with partnership satisfaction in heterosexual intimate couples (27). On that basis we might hypothesize that due to their more adaptive personality traits LKD have a higher partnership satisfaction compared to the general population. However, partnership satisfaction as well as life events are known to effect personality characteristics (28, 29), hampering causal interpretations.

Additionally, studies have shown that being in a happy relationship has a positive impact on the individual's physical and mental health (11). As already described above LKD are known to be “healthier” than the general population (23). It can be assumed that there exists a reciprocal effect in that “healthy” individuals are more often involved in a partnership and at the same time benefit from high partnership satisfaction with an improvement of their health status.

Furthermore we compared the partnership satisfaction between spouse donors and non-spouse donors. 23.1% of the spouse donors and 18.7% of the non-spouse donors perceived their partnership as improved since the time of living donor transplantation. In both groups very few unhappy relationships defined by a QMI-D score below 34 were found. In the literature conflicting results regarding this comparison have been reported. Terasaki et al. (8) surveyed 176 living spousal kidney donors of which 47% reported an improved partnership satisfaction after transplantation. Also the recipients reported an improved relationship (8). In contrast, Serur et al. (16) examined 11 spouse donors and 31 non-spouse donors in their study using the Revised Dyadic Adjustment Scale and found a higher satisfaction in couples in which the spouse was not the recipient of the kidney compared to couples after donation to the spouse.

Interestingly, female spouse donors representing 69.2% of all spouse donors in our sample reported a significantly higher QMI-D score than female non-spouse donors. This was not found for male donors. First of all it seems important to take a look at the gender imbalance in spouse donors. It is a well-known phenomenon that more female donors are donating to their male partner than the other way around (30). One obvious reason is the fact that men have a higher life risk for ESRD and suffer more frequently from ESRD than women (31, 32). Another explanation might be that the male partner is oftentimes the main wage earner of the family. The requested 6 week period of low physical activity might not be compatible to the working circumstances of male partners especially when occupied in building and construction industry. In addition, men are statistically more affected by cardiovascular diseases and other preconditions, which might exclude them as LKD (31, 33).

To understand the differences in partnership satisfaction between female spouse donors and non-spouse donors it seems to be important to understand that socioeconomic and cultural factors have an influence on people's motivation and decision to donate (30, 31). Von Zur-Mühlen et al. (30) assessed the motives of LKD: Women wanted to help the recipient whereas men described feeling a moral obligation to donate. After the donation significantly more female donors described experiencing a “positive impact” on their lives (30). As the item has not been further specified, we can only speculate wherein the impact lies. It can be hypothesized that a high partnership satisfaction can be seen as a positive development. Additionally, it seems possible that through the evaluation process especially women with already high partnership satisfaction are chosen as donors. Therefore, further research is needed to gain better understanding of the factors which influence partnership satisfaction after living kidney donation. In addition, a comparison to couples of post-mortal organ donation might be of interest. It might be possible that the successful transplantation itself improves the partnership satisfaction due to more flexibility in daily life and better health of the recipient (e.g., of a spouse or a child living in the same household). Hemodialysis (HD) is the most frequent renal replacement therapy in Germany and usually dialysis sessions of 4–6 h three times weekly are required. Chronic HD patients oftentimes suffer from physical impairment, fatigue and depression which could also affect partnership satisfaction. At the time of transplantation the recipients either have an advanced stage of chronic kidney disease with eGFR below 15 ml/min or are on dialysis (34) thus the improvement of the recipient's health most likely positively affects also the partnership dynamics.

Most prior work suggests that there is a positive or no change at all in the relationship between donor and recipient after the donation (8, 13, 14). Our results are in good agreement with former findings: 97.1% of our donors report an “improved” or “unchanged” relationship with the recipients. Comparing spouse to non-spouse donors no significant difference could be found. As already described above potential LKD have to undergo an evaluation process in which great regard is put on the relationship between donor and recipient. In Germany living donation is only allowed when a close emotional relationship between donor and recipient is proven which is particularly true for spouses, children, other relatives, or very close and longstanding friends. Due to the German Transplantation Law non-directed altruistic kidney donation is not permitted. These circumstances lead to a special selection of donors with a good relationship to the recipient before the transplantation (13), which seems to endure the transplantation as there are only few donors reporting a deterioration of the relationship. From a clinical viewpoint, however, special attention should be paid to identifying those donors experiencing (psychosocial) difficulties and a deterioration of partnership satisfaction after transplantation.

Yet, some limitations are worth noting. Even after contacting the donors up to three times, we only got response from 60%. It might be possible, that those responding to the survey had a more positive experience after donation than those not responding. The non-participants were significantly older with a longer time period since donation. However, there was no difference regarding sex.

Additionally there were significant differences regarding sociodemographic variables between the population sample and the LKD sample. As the purpose of the population sample is to be representative for the general population, differences with the LKD sample had to be accepted. It has been known before that the general population does not present the ideal control group as LKD are particularly “healthy” (23). However, the differences found in our study remained significant after adjusting for age, sex, and educational level.

Furthermore, there are no baseline data available as the QMI-D was not assessed before transplantation. In addition, partnership satisfaction was estimated based on the donor's QMI-D score. Eventually, the evaluation of the donors differs from the evaluation of the partner. Future research should explore partnership satisfaction by evaluating the donor as well as the partner and assess the pre-donation as well as the post-donation period.

## Conclusion

In conclusion, the findings of our study suggest that LKD are more frequently living in a partnership than individuals from the general population. The partnership satisfaction evaluated with the QMI-D is significantly higher in the donor sample compared to the population sample. In addition, less unhappy partnerships defined by a QMI-D score below the cut-off can be found in the donor sample. Above that, spouse donors, especially women having donated to their partner, experience even higher partnership satisfaction than non-spouse donors. These results show that LKD experience above average partnership satisfaction. Due to the cross-sectional design of our study no baseline data are available regarding partnership satisfaction before donation. However, when asked about a change in the relationship with the recipient more than 97% reported an unchanged or improved relationship. Our findings are consistent with previous studies showing that living kidney donation does not appear to negatively impact relationship satisfaction in most donors. Future studies with a longitudinal design are required to confirm these results. Assessing the donor's as well as the partner's perspective might be worthwhile.

## Author contributions

MdZ, KW, and FG designed the study and received funding. IP and MM were mainly responsible for data acquisition. MdZ and MN analyzed the data and MN wrote the first draft. All authors contributed significantly to the interpretation of the data and the final version of the manuscript. All authors gave final approval of the version to be published.

### Conflict of interest statement

The authors declare that the research was conducted in the absence of any commercial or financial relationships that could be construed as a potential conflict of interest. The reviewer FV declared a past collaboration with the authors MN and MdZ to the handling Editor.

## References

[1] Wyld M Morton RL Hayen A Howard K Webster AC. A systematic review and meta-analysis of utility-based quality of life in chronic kidney disease treatments. PLoS Med. (2012) 9:9. 10.1371/journal.pmed.1001307 22984353 PMC3439392

[2] Deutsche Stiftung Organtransplantation Niedrigster Stand der Organspenden Seit 20 Jahren. (2018). Available online at: https://www.dso.de/dso-pressemitteilungen/einzelansicht/article/niedrigster-stand-der-organspenden-seit-20-jahren.html

[3] Deutsche Stiftung Organtransplantation Grafiken zur Organspende und -transplantation in Deutschland. (2018). Available online at: https://www.dso.de/presse/pressebilder-und-grafiken.html

[4] Meier-Kriesche HU Kaplan B. Waiting time on dialysis as the strongest modifiable risk factor for renal transplant outcomes: a paired donor kidney analysis. Transplantation (2002) 74:1377–81. 10.1097/01.TP.0000034632.77029.91 12451234

[5] Deutsche Stiftung Organtransplantation Niere – Warteliste und Vermittlung. (2018). Available online at: https://www.dso.de/organspende-und-transplantation/warteliste-und-vermittlung/niere.html

[6] Lo P Sharma A Craig JC Wyburn K Lim W Chapman JR. Preconditioning therapy in ABO-incompatible living kidney transplantation: a systematic review and meta-analysis. Transplantation (2016) 100:933–42. 10.1097/TP.0000000000000933 26425876

[7] Yoon HE Song JC Hyoung BJ Hwang HS Lee SY Jeon YJ. Comparison of long-term outcomes between spousal transplants and other living unrelated donor transplants: single-center experience. Nephron Clin Pract. (2009) 113:c241–9. 10.1159/000235248 19684408

[8] Terasaki PI Cecka JM Gjertson DW Cho YW. Spousal and other living renal donor transplants. Clin Transpl. (1997) 269–84. 9919411

[9] Gee CB Howe GW Kimmel PL. Couples coping in response to kidney disease: a developmental perspective. Semin Dial. (2005) 18:103–8. 10.1111/j.1525-139X.2005.18205.x 15771653

[10] Daneker B Kimmel PL Ranich T Peterson RA. Depression and marital dissatisfaction in patients with end-stage renal disease and in their spouses. Am J Kidney Dis. (2001) 38:839–46. 10.1053/ajkd.2001.27704 11576888

[11] Carr D Freedman VA Cornman JC Schwarz N. Happy marriage, happy life? Marital quality and subjective well-being in later life. J Marriage Fam. (2014) 76:930–48. 10.1111/jomf.12133 25221351 PMC4158846

[12] Proulx CM Helms HM Buehler C Marital quality and personal well-being: a meta-analysis. J Marriage Fam. (2007) 69:576–93. 10.1111/j.1741-3737.2007.00393.x

[13] Clemens KK Thiessen-Philbrook H Parikh CR Yang RC Karley ML Boudville N. Psychosocial health of living kidney donors: a systematic review. Am J Transplant. (2006) 6:2965–77. 10.1111/j.1600-6143.2006.01567.x 17294524

[14] Clemens K Boudville N Dew MA Geddes C Gill JS Jassal V. The long-term quality of life of living kidney donors: a multicenter cohort study. Am J Transplant. (2011) 11:463–9. 10.1111/j.1600-6143.2010.03424.x 21342446

[15] Neuhaus TJ Wartmann M Weber M Landolt MA Laube GF Kemper MJ. Psychosocial impact of living-related kidney transplantation on donors and partners. Pediatr Nephrol. (2005) 20:205–9. 10.1007/s00467-004-1749-9 15627165

[16] Serur D Charlton M Bretzlaff G Sinacore J Christos P Gordon-Elliott J. Is donating a kidney to a friend bad for your marriage? Nephrology (2015) 20:434–6. 10.1111/nep.12426 25900385

[17] Zimmermann T Lause M Heinrichs N Fragebogen zur Partnerschaftsqualität: quality of Marriage Index - Deutsche Version (QMI-D). Verhaltenstherapie (2015) 25:51–3. 10.1159/000371478

[18] Norton R Measuring marital quality: a critical look at the dependent variable. J Marriage Fam. (1983) 45:141–51.

[19] Khattak MW Sandhu GS Woodward R Stoff JS Goldfarb-Rumyantzev AS. Association of marital status with access to renal transplantation. Am J Transplant. (2010) 10:2624–31. 10.1111/j.1600-6143.2010.03318.x 21070605

[20] Robards J Evandrou M Falkingham J Vlachantoni A. Marital status, health and mortality. Maturitas (2012) 73:295–99. 10.1016/j.maturitas.2012.08.007 23007006 PMC3635122

[21] Waldron I Hughes ME Brooks TL. Marriage protection and marriage selection-prospective evidence for reciprocal effects of marital status and health. Soc Sci Med. (1996) 43:113–23. 10.1016/0277-9536(95)00347-9 8816016

[22] Tatangelo G McCabe M Campbell S Szoeke C. Gender, marital status and longevity. Maturitas (2017) 100:64–9. 10.1016/j.maturitas.2017.03.002 28539178

[23] Wirken L van Middendorp H Hooghof CW Rovers MM Hoitsma AJ Hilbrands LB. The course and predictors of health-related quality of life in living kidney donors: a systematic review and meta-analysis. Am J Transplant. (2015) 15:3041–54. 10.1111/ajt.13453 26414703

[24] Lentine KL Kasiske BL Levey AS Adams PL Alberu J Bakr MA KDIGO clinical practice guideline on the evaluation and care of living kidney donors. Transplantation (2017) 101:1–109. 10.1097/TP.0000000000001769 PMC5540357 28742762

[25] de Zwaan M Erim Y Greif-Higer G Kröncke S Burgmer M Vitinius F. Ergebnisse einer repräsentativen Befragung zur Durchführung der psychosozialen Begutachtung vor Lebendnierenspende in Deutschland. Psychother Psychosom Med Psychol. (2017) 67:240–44. 10.1055/s-0043-102180 28722099

[26] Pollmann I Gueler F Mikuteit M Nöhre M Richter N Weissenborn K. Adaptive personality traits and psychosocial correlates among living kidney donors. Front Psychiatry (2017) 8:210. 10.3389/fpsyt.2017.00210 29109691 PMC5660284

[27] Malouff JM Thorsteinsson EB Schutte NS Bhullar N Rooke SE The five-factor model of personality and relationship satisfaction of intimate partners: a meta-analysis. J Res Pers. (2010) 44:124–7. 10.1016/j.jrp.2009.09.004

[28] Scollon CN Diener E. Love, work, and changes in extraversion and neuroticism over time. J Pers Soc Psychol. (2006) 91:1152–65. 10.1037/0022-3514.91.6.1152 17144771

[29] Jeronimus BF Ormel J Aleman A Penninx BW Riese H. Negative and positive life events are associated with small but lasting change in neuroticism. Psychol Med. (2013) 43:2403–15. 10.1017/S0033291713000159 23410535

[30] von Zur-Mühlen B Yamamoto S Wadström J. Few gender differences in attitudes and experiences after live kidney donation, with minor changes over time. Ann Transplant. (2017) 22:773–79. 10.12659/AOT.906129 29284769 PMC6248298

[31] Puoti F Ricci A Nanni-Costa A Ricciardi W Malorni W Ortona E. Organ transplantation and gender differences: a paradigmatic example of intertwining between biological and sociocultural determinants. Biol Sex Differ. (2016) 7:35. 10.1186/s13293-016-0088-4 27471591 PMC4964018

[32] Turin TC Tonelli M Manns BJ Ahmed SB Ravani P James M Lifetime risk of ESRD. J Am Soc Nephrol. (2012) 9:1569–78. 10.1681/ASN.2012020164 PMC3431421 22904351

[33] Piccoli GB Alrukhaimi M Liu ZH Zakharova E Levin A World Kidney Day Steering Committee. Women and kidney disease: reflections on world kidney day 2018. J Ren Care (2018) 44:3–11. 10.1111/jorc.12232 29405643

[34] Eriksson D Goldsmith D Teitsson S Jackson J van Nooten F. Cross-sectional survey in CKD patients across Europe describing the association between quality of life and anaemia. BMC Nephrol. (2016) 17:97. 10.1186/s12882-016-0312-9 27460779 PMC4962379

